# Comparative study on gene set and pathway topology-based enrichment methods

**DOI:** 10.1186/s12859-015-0751-5

**Published:** 2015-10-22

**Authors:** Michaela Bayerlová, Klaus Jung, Frank Kramer, Florian Klemm, Annalen Bleckmann, Tim Beißbarth

**Affiliations:** Department of Medical Statistics, University Medical Center Göttingen, 37099 Göttingen, Germany; Department of Hematology and Medical Oncology, University Medical Center Göttingen, 37099 Göttingen, Germany

**Keywords:** Gene set analysis, Pathway topology, Enrichment methods, Simulations, Accuracy, Sensitivity

## Abstract

**Background:**

Enrichment analysis is a popular approach to identify pathways or sets of genes which are significantly enriched in the context of differentially expressed genes. The traditional gene set enrichment approach considers a pathway as a simple gene list disregarding any knowledge of gene or protein interactions. In contrast, the new group of so called pathway topology-based methods integrates the topological structure of a pathway into the analysis.

**Methods:**

We comparatively investigated gene set and pathway topology-based enrichment approaches, considering three gene set and four topological methods. These methods were compared in two extensive simulation studies and on a benchmark of 36 real datasets, providing the same pathway input data for all methods.

**Results:**

In the benchmark data analysis both types of methods showed a comparable ability to detect enriched pathways. The first simulation study was conducted with KEGG pathways, which showed considerable gene overlaps between each other. In this study with original KEGG pathways, none of the topology-based methods outperformed the gene set approach. Therefore, a second simulation study was performed on non-overlapping pathways created by unique gene IDs. Here, methods accounting for pathway topology reached higher accuracy than the gene set methods, however their sensitivity was lower.

**Conclusions:**

We conducted one of the first comprehensive comparative works on evaluating gene set against pathway topology-based enrichment methods. The topological methods showed better performance in the simulation scenarios with non-overlapping pathways, however, they were not conclusively better in the other scenarios. This suggests that simple gene set approach might be sufficient to detect an enriched pathway under realistic circumstances. Nevertheless, more extensive studies and further benchmark data are needed to systematically evaluate these methods and to assess what gain and cost pathway topology information introduces into enrichment analysis. Both types of methods for enrichment analysis require further improvements in order to deal with the problem of pathway overlaps.

**Electronic supplementary material:**

The online version of this article (doi:10.1186/s12859-015-0751-5) contains supplementary material, which is available to authorized users.

## Background

Analysis of gene expression experiments typically yields long lists of differentially expressed genes (DEGs) [1]. To ease interpretation of such results it has been proposed to analyse expression data in the context of biological pathways or other biologically meaningful gene sets instead of individual genes [2]. There are many tests, which aim to detect pathways significantly enriched between two experimental conditions [3–6]. These tests were implemented into a plethora of methods that differ in many aspects ranging from null hypothesis formulation, through statistical framework up to pathway data encoding, database support and software availability.

We were interested to evaluate different methods for analysing gene expression data in the context of signalling pathways, with a special focus on comparing methods with different pathway representation strategies. There are different approaches how to handle a pathway in enrichment analysis. The traditional approach considers a pathway as a simple gene list omitting any knowledge of the gene and protein interactions. Methods belonging to this category are commonly called gene set (GS) analysis methods. Another way of integrating a pathway into enrichment analysis takes into account its graphical structure and/or topological measures. One of the first pathway topology-based (PT-based) methods was impact analysis proposed by Draghici et al. [7]. Since then this has approach become very popular, resulting in a number of PT-based algorithms being published [8–11].

Enrichment methods can be divided into two groups based on the null hypothesis: (i) competitive methods which are naturally linked with gene sampling for *p*-value calculation and (ii) the group of self-contained methods which is associated with subject sampling [12]. The basic difference between these two groups is that competitive methods compare genes in a pathway to its complement usually represented by the rest of the genes measured in the experiment, while self-contained methods consider only genes within a pathway and test their association with the phenotype. Both approaches have their limitations. On the one hand, competitive methods coupled with a gene sampling model for *p*-value calculation assume independence of genes, which is known not to be true. On the other hand, self-contained methods were criticized for being too powerful and thus yielding too many significant gene sets. Also the number of replicates in the experiments is often too low for subject sampling [12].

Another classification of enrichment methods separates them into over-representation analysis (ORA) and functional class scoring (FCS) approaches [13]. ORA is commonly referring to 2×2 table methods such as Fisher’s exact test, hypergeometric test and chi-squared test. It represents exclusively the competitive approach. The ORA methods require a strict cut-off in the list of DEGs and therefore the results are strongly dependent on the chosen threshold. FCS comprises methods, which first score genes and then transform gene level scores into pathway level scores. This group includes both competitive and self-contained methods, depending on the pathway-level transformation and significance assessment of pathway level score. Most of the PT-based methods can be classified into either ORA or FCS, however in some cases it might be difficult to draw a strict line.

From the user’s point of view there are several features of the enrichment methods to be considered besides classification of the methods according to methodological and statistical framework. For instance, the implementation and availability of the software can determine how popular a method becomes. Furthermore, several pathway databases such as Kyoto Encyclopedia of Genes and Genomes (KEGG) [14], Reactome [15], Pathway Interaction Database [16] or BioCarta [17] make data available for pathway analysis. While it is rather straightforward to supply simple gene set of a pathway for GS methods, providing a pathway with topology information and integrate it into a PT-based method can be more difficult. Therefore, the utility of PT-based methods also depends on their support of different pathway databases.

Several studies comparing different GS analysis methods were already published [18–20], as well as review papers offering surveys of PT-based methods [13, 21]. However, to our best knowledge there is no paper comparing the GS against the PT-based approach systematically.

Therefore, the aim of our study is to evaluate GS versus PT-based approaches using simulated gene expression data as well as benchmark datasets. For comparability of the methods we focused mainly on competitive ones, including three GS tests and four PT-based methods implemented in the R statistical computing environment (see Table 1). These seven methods represent both ORA and FCS approaches.

**Table 1:** Summary of seven enrichment methods evaluated in this study

Test/Method name	Pathway representation	ORA/ FCS	R-function/ package version	Database support	Null hypothesis
Wilcoxon rank sum	GS	FCS	wilcox.test	Any input	Competitive
Kolmogorov-Smirnov	GS	FCS	ks.test	Any input	Competitive
Fisher’s exact	GS	ORA	fisher.test	Any input	Competitive
SPIA	PT-based	ORA-like	2.12.0.	KEGG	Competitive
CePa ORA	PT-based	ORA-like	0.5.	KEGG, Reactome, NCI-Nature, BioCarta	Competitive
CePa GSA	PT-based	FCS-like	0.5.		Self-contained
PathNet	PT-based	unclass.	1.3.0.	KEGG	Competitive

Methods were classified according to the representation of a pathway into the gene set (GS) enrichment and the pathway topology-based (PT-based) methods. Second column stratifies the methods into over-representation analysis (ORA) and functional class scoring (FCS). However, for PT-based methods this classification is not always explicit, therefore we used ‘-like’ suffix and ‘unclass.’ for an unclassified method. Third column shows the utilized R-functions for GS methods and versions of R-packages of PT-based methods. Next column summarizes the pathways databases which are provided within the R-packages. According to the null hypothesis most of the methods are competitive, only the GSA variant of the CePa method is self-contained

To perform a fair comparison of the different methods we faced two major challenges: First, it was necessary to provide the same pathway data inputs for all methods, which was in particular challenging for PT-based methods. The integration of topological pathway data is described in detail in the third section of the *Methods*. Second, to simulate expression data for evaluation of PT-based methods was a comprehensive problem, which resulted in a complex simulations scheme. Hence, we described *Simulations* as a separate chapter.

## Methods

In this chapter we present in detail 3 GS and 4 PT-based enrichment methods and their use within this work. Further, we describe how pathway data were processed and integrated into the methods. The last section of the *Methods* introduces the analysis of benchmark data.

### Gene set enrichment methods

We chose rather basic statistical tests to represent the GS analysis approach: Wilcoxon rank sum (WRS), Kolmogorov-Smirnov (KS) and Fisher’s exact (FE) tests. These tests were implemented in various flavours and extensions in multiple tools. For instance, the popular GSEA [4] is based on KS statistic, the SAFE [6] and CAMERA [22] tools employ the WRS test in different fashions and the FE test is implemented in software packages such as GOStat [5] and Onto-Express [3].

We utilized the R-functions *wilcox.test*, *ks.test* and *fisher.test* to perform WRS, KS and FE analysis, respectively within the R statistical language and environment [23], version 2.15.1. All three tests are competitive gene sets approaches, which require a list of *p*-values for differential expression [2]. WRS and KS are FCS methods transforming the list of *p*-values into ranks. In our setting, WRS tests whether the distribution of ranks of the genes in a set is shifted to the left from a distribution of ranks of the genes in corresponding complement to the gene set. KS test compares the ranks of genes in a set to a uniform distribution.

The FE test is based on ORA. The cut-off in the list of differentially expressed genes (DEGs) was defined as false discovery rate below 0.05 (FDR <0.05). FE is testing independence of rows and columns in 2x2 contingency table (while the margins are fixed) and *p*-values are directly obtained using hypergeometric distribution.

### Pathway-topology based enrichment methods

In this study we evaluated PT-based algorithms from three R-packages: *SPIA*, *CePa* and *Pathnet.* Two variants of the CePa method were implemented in the *CePa* R-package, the so-called CePa ORA and CePa GSA, which we consider as two distinct methods.

#### SPIA

Signaling pathway impact analysis (SPIA) is an enrichment method combining two types of evidence, which are represented by two *p*-values [8]. The first *p*-value originates from a simple ORA, which assumes that the number of DEGs in a given pathway follows hypergeometric distribution. The second, so-called perturbation *p*-value is computed in several steps and incorporates pathway topology information. To obtain the perturbation *p*-value, first, for each gene in a pathway a perturbation factor is computed. It captures the logarithm of the fold-change (logFC) of a gene and the sum of perturbation factors of the direct upstream genes of a gene normalized by the number of all downstream genes. Each term of the sum is weighed by type of interaction between the genes: 1 and −1 for activation and inhibition, respectively. This results in upstream gene influencing perturbation factors of many downstream genes. In a second step the perturbation accumulation at the level of each pathway gene is calculated. It is defined as the difference between the gene perturbation factor and its observed logFC. Finally, the total pathway accumulated perturbation is computed as a sum of the accumulated perturbations of pathway’s genes. Significance is assessed in a bootstrap procedure, resulting in a perturbation *p*-value. The two *p*-values are then combined into a global *p*-value for each pathway using Fisher’s product test.

#### CePa ORA and CePa GSA

CePa [24] is a weighed gene set analysis approach in which weights are assessed by network centralities. The CePa ORA method weighs the nodes of DEGs according to one of 5 centrality measures and then sums them up to the pathway level score. To obtain a *p*-value the null distribution of the pathway score is generated by permuting the DEGs on a given pathway topology. The centrality measure options are in- and out-degree, betweenness and in- and out-largest reach. Degree centrality measures the number of nodes directly connected to the given node, betweenness refers to the number of information streams passing through a given node and largest reach quantifies how far a node can send or receive information. Along 5 centrality options CePa also calculates an equal weight model where all weights are set up to 1. Therefore, six *p*-values for each pathway are calculated. We followed the authors’ recommendation to try every centrality option in the search for significant pathways. Hence, the smallest out of 6 *p*-values was selected to represent pathway significance.

Furthermore, the CePa method proposed a node-based instead of a gene-based ID mapping approach. That means, if any member of a complex or a group of genes residing in one node is differentially expressed then the node is considered as differentially expressed. Also nodes representing non-gene components of a pathway such as microRNA and small molecules are retained in the pathway topology. However, these last two features of the CePa algorithm were suppressed using our pathway input data (for details see *Pathway data* section).

CePa GSA performs self-contained univariate gene set analysis where node level statistics are weighted by centrality measures and then transformed into pathway level statistics. CePa GSA implements several alternatives for node level statistics and transformation functions, however, we kept the default options: for node level scores the absolute value of t-statistic was used and for computing pathway level statistics mean was used as default transformation function. Then the significance of each pathway is assessed by permuting sample class labels.

#### PathNet

PathNet [11] is an enrichment method combining 2 types of evidences on gene-level in contrast to SPIA which combines 2 types of evidences on pathway-level. This method considers so-called direct and indirect evidence. Direct evidence from expression data is represented by nominal *p*-values of the DEGs. Indirect evidence of a gene is calculated from direct evidences of its neighbours in a *pooled pathway*. The *pooled pathway* is a big network created by merging all pathways presented in a given database. To calculate indirect evidence, first, indirect evidence score of a gene is defined as a sum of the negative log10 transformed *p*-values of all its neighbours in the *pooled pathway*. Secondly, the null distribution of this score is reconstructed by randomizing direct evidence *p*-values on the *pooled pathway* with a fixed topology and a corresponding indirect evidence *p*-value is estimated. Finally, a *p*-value for each gene is obtained by aggregating direct and indirect evidence *p*-values using Fisher’s method. The significance of a pathway is assessed via a hypergeometric test.

### Pathway data

Real pathway data available from public sources were used in this work. Within R various approaches to integrate pathways into enrichment analyses are available [25]. The three R-packages of PT-based methods comprise default pathway data of KEGG and other databases (see Table 1) in a preprocessed and suitable format. However, to ensure comparability of the evaluated algorithms we supplied all methods with the same KEGG pathway data.

#### Parsing the KEGG database

**Fig. 1:**
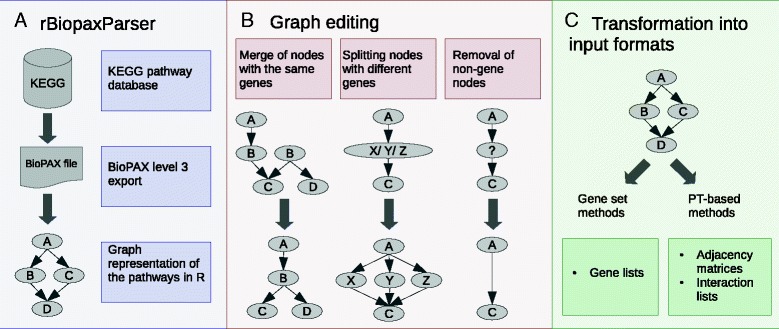
Pathway data input preparation. The workflow shows the three steps necessary to provide the same pathways to all methods. **a** Non-metabolic KEGG pathways were parsed into R and represented as directed interaction graphs. **b** The graphs were edited in order to affiliate a single graph node with a single gene ID. **c** The adjusted graphs were further transformed into suitable input formats for both, GS and PT-based methods

A BioPAX level 3 export of KEGG pathway database was downloaded on March 2013 and parsed into R using the *rBiopaxParser* R-package [26] (Fig. 1a). Non-metabolic pathways were selected and transformed into interaction graphs in which the nodes represent genes and the directed edges represent activation or inhibition processes between the genes.

After mapping HUGO Gene Nomenclature Committee (HGNC) gene symbol IDs on the graph nodes several editing steps had to be taken (Fig. 1b). First, in case that several nodes were annotated with the same gene symbol, these nodes were merged into a node, which shared all incoming and outgoing edges of the original nodes. Next, protein complexes or gene families often occupied a single node resulting in multiple symbol IDs embedded in this node. Such a node was split into multiple nodes and each one was assigned with a single gene symbol. Finally, nodes representing small molecules and other non-gene components were removed in a fashion that the parents and children of such a node stayed connected.

#### Transformation into suitable inputs

Depending on the method, suitable pathway input has to be provided implying graphs transformations [27]. For GS methods pathway graphs were converted into simple lists of genes. However, for the PT-based methods specific pathway topology inputs were required (Fig. 1c).

To create the SPIA pathway input, a graph of each pathway was transformed into a list of 2 adjacency matrices for activation and inhibition processes. Accordingly, a vector of weights β was set to β = {1, −1} to reflect activation and inhibition. For CePa a pathway catalogue was constructed comprising a list of pathways with the interaction IDs, and a table with columns for the interaction IDs and for the corresponding input and output interaction components. The pathway catalogue also included a mapping table. However, in our pathway data a single gene ID was always mapped onto one node. Input for PathNet consisted of an adjacency matrix of a pooled pathway, which was created by merging all database pathways, an interaction table with pathway IDs and a mapping table. The mapping table was required as PathNet algorithm transforms gene IDs into numeric objects and artificial integer IDs had to be generated.

All pathway data inputs were regenerated for the second simulation study (see *Simulations* chapter). In this study pathway graph nodes were relabelled with new synthetic IDs to construct non-overlapping pathways with unique components, while the topology of the pathway remained intact.

### Benchmark data

All datasets from the *KEGGdzPathwaysGEO R-package* and 12 datasets from the *KEGGandMetacoreDzPathwaysGEO* R-packages were used as benchmark data [18, 28]. A total of 36 publicly available disease datasets (see Table 2) were analysed in the same fashion: First, multiple probes per Entrez gene ID were removed by retaining the probe with the highest average expression. Next, the genes that could not be mapped onto any pathway were filtered out. Differential expression analysis was performed by fitting linear models using the empirical Bayes method as implemented in the *limma* R-package [29] and *p*-values were adjusted for multiple testing using the method of Benjamini & Hochberg [30]. Each of the 36 datasets was matched with the corresponding KEGG pathway according to its name, e.g. a dataset of colon cancer patients was associated with the colorectal cancer pathway. Such a pathway was then called a target pathway and its *p*-value and rank in the database were further evaluated [18, 28].

**Table 2:** Summary of 36 datasets used in benchmark analysis

GEO accession	Disease/Target pathway	Samples	Ref.
GSE781	Renal cell carcinoma	17	[36]
GSE1297	Alzheimer’s disease	16	[37]
GSE3467	Thyroid cancer	18	[38]
GSE3585	Dilated cardiomyopathy	12	[39]
GSE3678	Thyroid cancer	14	---
GSE4107	Colorectal cancer	22	[40]
GSE5281_EC	Alzheimer’s disease	21	[41]
GSE5281_HIP	Alzheimer’s disease	23	[41]
GSE5281_VCX	Alzheimer’s disease	31	[41]
GSE6956AA	Prostate cancer	10	[42]
GSE6956C	Prostate cancer	16	[42]
GSE8671	Colorectal cancer	64	[43]
GSE8762	Huntington’s disease	22	[44]
GSE9348	Colorectal cancer	82	[45]
GSE9476	Acute myeloid leukemia	63	[46]
GSE14762	Renal cell carcinoma	21	[47]
GSE15471	Pancreatic cancer	70	[48]
GSE16515	Pancreatic cancer	30	[49]
GSE18842	Non-small cell lung cancer	88	[50]
GSE19188	Non-small cell lung cancer	153	[51]
GSE19728	Glioma	21	[52]
GSE20153	Parkinson’s disease	16	[53]
GSE20291	Parkinson’s disease	33	[54]
GSE21354	Glioma	17	[52]
GSE1145	Dilated cardiomyopathy	26	---
GSE14924_CD4	Acute myeloid leukemia	20	[55]
GSE14924_CD8	Acute myeloid leukemia	21	[55]
GSE16759	Alzheimer’s disease	8	[56]
GSE19420	Type II diabetes mellitus	24	[57]
GSE20164	Parkinson’s disease	11	[53]
GSE23878	Colorectal cancer	38	[58]
GSE24739_G0	Chronic myeloid leukemia	12	[59]
GSE24739_G1	Chronic myeloid leukemia	12	[59]
GSE32676	Pancreatic cancer	32	[60]
GSE4183	Colorectal cancer	23	[61]
GSE7305	Endometrial cancer	20	[62]
Total:		1127	

The benchmark datasets were retrieved from *KEGGandMetacoreDzPathwaysGEO* and *KEGGdzPathwaysGEO* R-packages. The columns of summary table represent the accession number from GEO database, the name of target pathway, the number of samples for each dataset and the reference of original publication

## Simulations

**Fig. 2:**
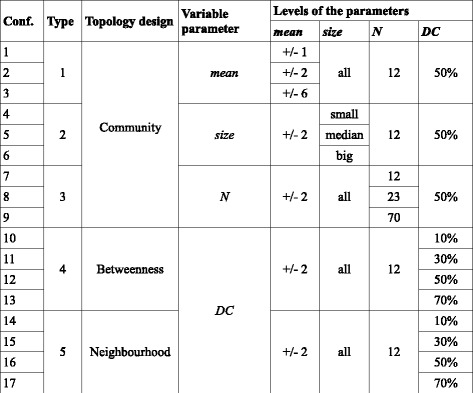
Five simulation types with 17 different parameter configurations. Simulation types comprise different combinations of a topology design and a variable parameter. Within one type sever al levels of one parameter were explored while the levels of the other parameters were fixed. This setting resulted in 17 distinct configurations (Conf.) of the parameters

We generated synthetic expression data for two simulation studies each comprising 5 simulation types. Simulation types differed in the topology designs for pathway deregulation and in a choice of the parameter whose ranges were investigated (see Fig. 2).

The two distinct simulation studies differed in their gene ID annotation. While in the first study real gene HGNC symbols were used, in the second study these symbols were replaced by unique synthetic IDs for all nodes of all pathways in order to prevent overlapping of the pathways.

In both studies expression data were drawn from a multivariate normal distribution, always consisting of 10 control and 10 treatment samples. As treatment samples we refer to the group in which we introduced changes in the *p*-dimensional mean vector. By introducing changes in the mean vector we controlled expression levels of the genes reflecting fold-changes between the two groups. The genes that had an increased expression above 0 are called affected. In the (*p* × *p)-*dimensional covariance matrix employed for the multivariate normal distribution, we introduced a correlation of 0.8 between genes in the same pathway and 0.05 otherwise (see Additional file 1B). Variance in the matrix was set to 2. The same covariance matrix was used for the generation of both treatment and control samples.

### Simulation studies

The differences between simulation study 1 and 2 originate from their different pathway data inputs.

#### Study 1: original pathways with overlapping genes

When generating expression data for the original pathway simulation study the dimension of mean vector was set to *p* = 3173. This number represents the total number of unique gene symbols in our KEGG pathway graphs. As the genes in this study can belong to multiple pathways the covariance matrix was not positive-definite (see Additional file 1A). We computed its close positive-definite approximation using the *sfsmisc* R-package (function *posdefify*).

#### Study 2: non-overlapping pathways with unique gene IDs

To simulate expression data in the second study the mean vector dimension was *p* = 7697. This number represents the total number of nodes in our KEGG pathway graphs. The covariance matrix of *p* × *p* was constructed in a similar manner as in the first study, but as pathways do not overlap the matrix was positive-definite by default.

### Variable parameters

In each study we performed 5 simulation types. Within a single simulation type one of the 4 parameters was explored on several levels, while the other 3 parameters were fixed at a certain level (see Fig. 2). This resulted in 17 distinct configurations of parameters. Each parameter configuration was examined in 1000 simulation runs, meaning that 1000 expression matrices were generated for the given configuration.

The variable parameters are mean vector (*mean*), pathway size (*size*), number of pathway (*N*) and detection call (*DC*). The three levels of *mean* = {+/−1, +/−2, +/−6} reflect expression changes of the affected genes between control and treatment groups. The size of the deregulated pathways *size* = {small, medium, big} is given by the number of genes in a pathway. The number of deregulated pathways *N* = {12, 23, 70} represents different proportions out of all 116 pathways in the KEGG database. Four levels of the detection call *DC* = {10 %, 30 %, 50 %, 70 %} give the percentage of affected genes in a deregulated pathway. The levels of these parameters were adjusted to reflect ranges from small to extreme effects that can be found in real data. As the default – fixed parameter level we chose moderate levels or the most general setting, depending on a particular parameter. For a more detailed description of the variable parameters see Additional file 2.

### Topology designs for pathway deregulation

**Fig. 3:**
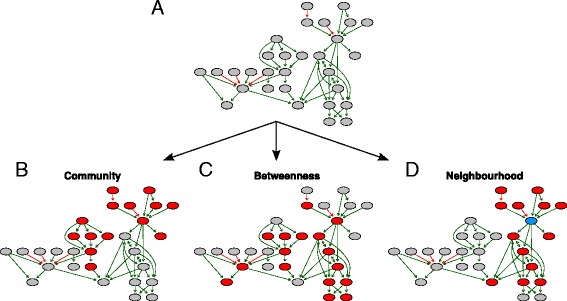
Topology designs for pathway deregulation. **a** Example of a particular pathway with 30 genes. In order to deregulate this pathway on detection call level e.g. *DC* = 50 % (+/− 5 %) we needed to assign 14–16 affected gene to this pathway and allocate them on the pathway graph according to 3 topology approaches. **b** In the community design two gene communities were selected to be affected (depicted in red). **c** Top scored betweenness genes were depicted in red. **d** Gene neighbourhood of order 2 of the blue gene was affected (in red). The colour coding of graph edges represents activation (green) and inhibition (red) interactions between the nodes

In order to deregulate a pathway within a stimulation we needed to affect some of its genes. We examined 3 approaches how to reflect pathway topology to allocate affected gene in a deregulated pathway: community, betweenness and neighbourhood approach (Fig. 3).

Communities in a given pathway were detected by an algorithm for greedy optimization of modularity implemented in the *igraph* R-package (*fastgreedy.community* function) [31]. It aims to find modules with dense connection between the module nodes and spare connections between nodes of different modules. For each pathway we searched for a community which represented 45-55 % detection call (DC). However, for some pathways several communities had to be joined or a too big community had to be cut to get the appropriate DC (Fig. 3b).

To select affected genes in the betweenness deregulation design we considered the top highest scored betweenness nodes (Fig. 3c). Betweenness of a node is defined as the number of all shortest paths in a directed graph going through a given node. The required number of the top scored nodes for the given DC was assigned as affected.

In the neighbourhood deregulation approach one node of a pathway is chosen and then all nodes within certain distance create the neighbourhood (Fig. 3d). In the search for the neighbourhoods representing different ranges of DC we calculated the neighbourhoods of several orders for each pathway nodes and chose the one best fitting the required DC level (+/− 5 %).

### Summary of a single simulation run

Within a given parameter configuration for a run, a certain number of pathways (depending on the setting) was randomly chosen to be deregulated by assigning affected genes. Then new expression data for this particular run were drawn from the multivariate normal distribution.

The expression matrix was directly supplied to the CePa ORA and CePa GSA algorithms. For the rest of the methods linear models from the *limma* package were fitted to identify differential genes with theirs logFC, *p*-values and FDRs (false discovery rates). Those were further supplied to the enrichment methods according to the specific requirements of each method.

All 7 methods were evaluated within each simulation run on the basis of the same expression as well as pathway input data.

## Results

### Parsed pathway data

We parsed 116 signalling pathways of the KEGG database into R using *rBiopaxParser* and transformed them into interaction graphs. These graphs comprised 7697 nodes in total, representing 3173 unique genes according to gene symbol IDs. On average a single gene was present in 2.4 pathways indicating the extent of pathway overlaps. The pathways comprised from 5 up to 380 genes with a median pathway size of 55.5 genes (see Additional file 3). The median number of connected components in a single pathway interaction graph was 2.

### Simulation results

In both simulation studies seven enrichment methods were evaluated in terms of accuracy, sensitivity and specificity. Here, sensitivity describes the proportion of true-positive pathways detected out of all pathways assigned as deregulated and specificity describes the proportion of true-negative pathways out of all pathways that were not deregulated (i.e. without assigned affected genes). The accuracy is then given as the proposition of true-positive and true-negative pathways detected by a method out of all 116 pathways. Within one study, for each of the 17 parameter configurations we computed the median accuracy, sensitivity and specificity of 1000 simulation runs. As in many scenarios 12 pathways was the maximum number of true-positives, the accuracy measure was mainly driven by true-negatives. Hence, it reflects the specificity measure to a bigger extent. Therefore, we focus mainly on accuracy and sensitivity as they provide more complementary view on the results and the specificity results are reported in Additional file 4.

#### Study 1 with original pathways (Fig. 4)

**Fig. 4:**
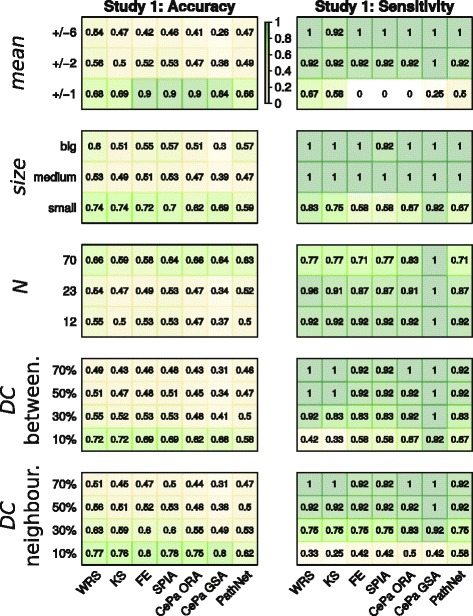
Accuracy and sensitivity in the simulation study 1: original pathways with overlapping genes. Accuracy and sensitivity scores of 7 methods under 17 parameter configurations. Each cell summarizes a median value of 1000 simulation runs and within each run 116 pathways were evaluated. The same colour code key implies for all simulation types. Wilcoxon rank sum test (WRS), Kolmogorov-Smirnov test (KS), Fisher’s exact test (FE)

With weak changes of the mean vector (*mean* = +/−1) sensitivity of ranking GS methods – 0.67 for WRS and 0.58 for KS – was the best, followed by PathNet with a sensitivity of 0.5. All methods based on ORA (both GS and PT-based) had 0 sensitivity, however, within the accuracy they reached 0.9. With a higher mean change, i.e. *mean* = {+/−2, +/−6}, all methods were comparably sensitive (between 0.92 and 1), while WRS reached the best accuracy scores of 0.56 and 0.54.

In the case of small pathways being deregulated all methods were less sensitive than for bigger pathways and CePa GSA (self-contained) and WRS performed best at this parameter level with a sensitivity of 0.92 and 0.83, respectively. However, within all methods the accuracy was highest for small pathways (ranged between 0.59–0.74) and decreases with larger pathways.

When exploring the effect of number of pathways which were deregulated, all methods were most accurate (0.58–0.66) when more than half of the database pathways were deregulated (*N* = 70) with WRS and CePa ORA performing the best.

At low level of detection call (*DC* = 10 %) coupled with betweenness topology design PT-based methods and FE test were more sensitive (0.58–0.92) than ranking GS methods (0.33–0.58). However, on 30 % and higher *DC* levels all methods performed comparably well in the term of sensitivity (0.83–1). At the same low level of detection call (*DC* = 10 %) coupled with neighbourhood topology design PathNet had the highest sensitivity of 0.58, followed by other PT-methods and FE (0.42–0.5). However, in both DC simulation types the accuracy ranged only between 0.56 and 0.31 for *DC* = {50 %, 70 %}.

The specificity and accuracy over most of the parameter configurations were very low for all methods (Fig. 4 and Additional file 4). Thus, we conducted second simulation study, in which we performed the same 5 simulation types on the non-overlapping pathways with unique gene IDs.

#### Study 2 with non-overlapping pathways (Fig. 5)

**Fig. 5:**
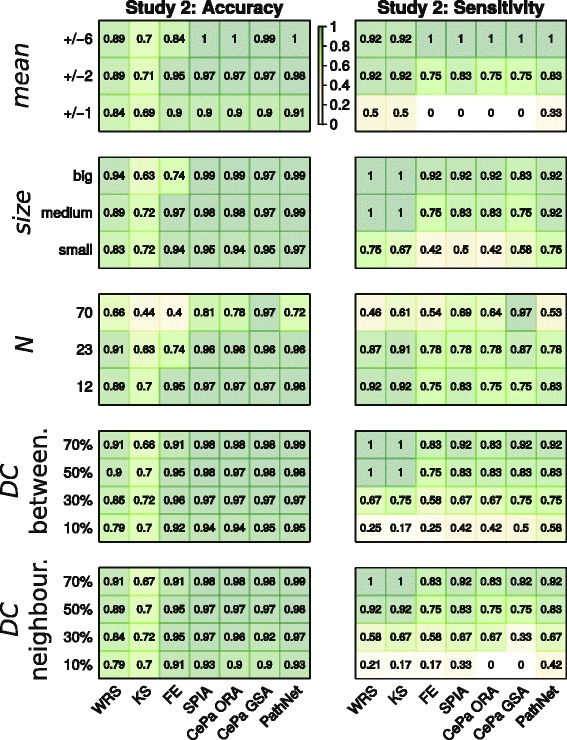
Accuracy and sensitivity in the simulation study 2: non-overlapping pathways with unique gene IDs. Accuracy and sensitivity scores of 7 methods under 17 parameter configurations. Each cell summarizes a median value of 1000 simulation runs and within each run 116 pathways were evaluated. The same colour code key implies for all simulation types. Wilcoxon rank sum test (WRS), Kolmogorov-Smirnov test (KS), Fisher’s exact test (FE)

Similarly to the results of study 1, GS methods based on ranking were the most sensitive (0.5) for the low level of *mean* parameter (+/−1). In detecting small pathways the best sensitivity (0.75) was reached by WRS and PathNet. However, the accuracy was the best for PT-based methods for most of the levels of both *size* and *mean* parameters. The sensitivity of all methods except CePa GSA decreased when many pathways were deregulated (*N* = 70), even to a bigger extent than in study 1. Interestingly, for *N* = 70 FE and KS accuracy was only 0.4 and 0.44, respectively, while the other methods reached an accuracy between 0.66 and 0.97. Both simulation types with DC parameter coupled with neighbourhood and betweenness topology designs showed similar behaviour patterns. When pathways did not overlap a DC level of 50 % was needed to achieve sensitivity over 0.75, whereas for the study with original pathways all methods reached 0.75 sensitivity at a *DC* = 30 % level. For the low DC (*DC* = 10 %) PathNet’s sensitivity was the best. In term of accuracy the PT-methods performed the best.

#### Overall accuracy, sensitivity and specificity in the two simulation studies

**Fig. 6:**
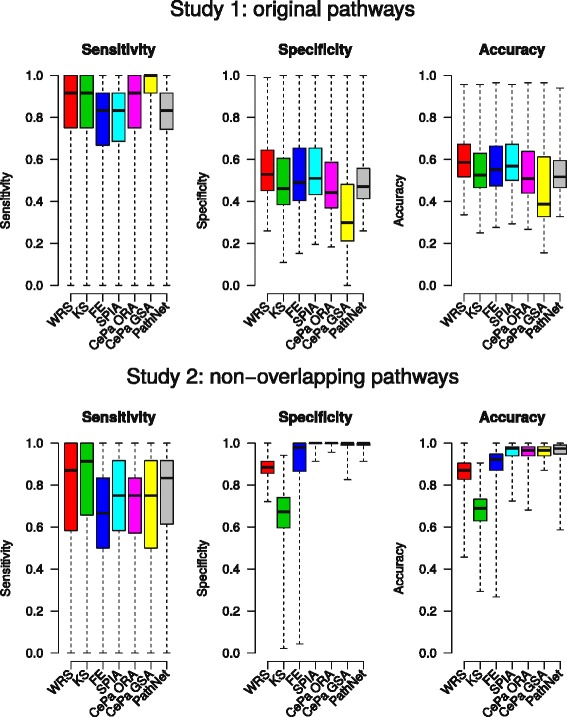
Overall sensitivity, specificity and accuracy in the simulations. Distribution of 3 measures for each method over 17000 simulation runs for study 1 with the original pathways with overlapping genes and study 2 with non-overlapping pathways with unique gene IDs

Distribution of accuracy, sensitivity and specificity results over all 17 simulation configurations was visualized for each method within the both studies (Fig. 6).

In the first simulation study with original overlapping pathways the best accuracy was achieved by WRS and SPIA. This was also reflected in the best specificity of these two methods, while the CePa GSA performed as the least specific method. However, the CePa methods reached the best sensitivity, followed by ranking tests WRS and KS.

In the second simulation study with non-overlapping pathways, both overall accuracy and specificity increased, especially for the PT-based methods. Within GS methods there were prominent differences with KS performing the worst. However, KS with WRS showed as the most sensitive methods.

### Benchmark results

Besides simulated expression data, 36 microarray datasets comprising together 1127 samples were used as a benchmark to compare 7 enrichment methods. Every dataset represents a certain disease and has been linked to a defined target pathway from the KEGG database (see Table 2): For instance, for the dataset comparing colon cancer samples with controls the target pathway is ‘Colon cancer pathway’. However, the target pathway is only one of several potential true positive pathways in a given dataset. Therefore, we evaluated neither sensitivity nor accuracy to avoid a bias caused by false negative pathways. Instead, we inspected *p*-values of the target pathways in 36 datasets and their ranks in the whole KEGG database, expecting the target pathways to be ranked close to the top [28].

**Fig. 7:**
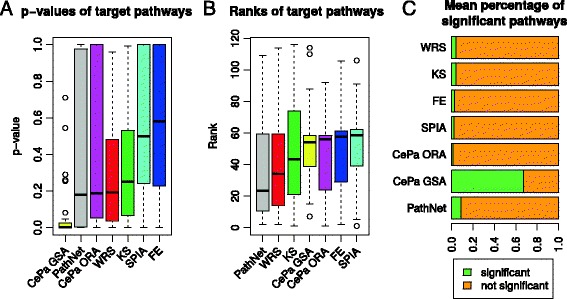
Comparison of 7 methods on the benchmark data. Distributions of *p*-values and ranks of the target pathways in 36 datasets. Methods are ordered according to the median *p*-value (**a**) and rank (**b**) from the best to the worst – lower values better performance. (**c**) Average percentage of the 116 pathways detected as significant and not significant by each method

The lowest *p*-values of the target pathways were detected by CePa GSA, followed by Pathnet, CePa ORA and WRS, all three with similar performance (Fig. 7a). In the ranking of the target pathways the PathNet method performed best (Fig. 7b). Figure 7c shows the proportion of the significant to the not significant pathways detected within the 36 datasets. The CePa GSA identified on average 67 % of all database pathways as significantly enriched, while for the rest of the methods it was between 1.5 % and 8.9 % of the significant pathways.

## Discussion

In order to place long lists of differential genes into a context of biological processes and pathways the enrichment analysis strategy is widely used. In this work we compared standard gene set (GS) enrichment tests with methods, which attempt to incorporate pathway topology information into analysis.

Comparing GS methods and pathways topology-based (PT-based) enrichment approaches is challenging in two regards. First, the pathway input data have to be corresponding for all methods to ensure a fair comparison. Therefore, we parsed a BioPAX export of the KEGG database and transformed the pathways into gene sets as well as various interaction graph representations for the different methods. Second, it is difficult to simulate expression data for PT-based methods. The signal in a pathway is mediated via multiple biological mechanisms. Capturing a pathway deregulation in a graph and reflecting this alteration in the synthetic expression data is an intriguing task. To our knowledge there is no coherent work published on this topic. We made one of the first attempts to generate expression data mirroring the topology of the deregulated pathways. However, there are several limitations in our simulation scheme. For instance, multivariate approaches usually require more complex covariance matrices for a fair and unbiased evaluation, therefore, we have not included multivariate self-contained methods.

We decided to use 3 approaches to allocate affected genes to a pathway: gene community, betweenness and neighbourhood. However, in a certain setting some methods might be favoured due to their inherent algorithms, e.g. both CePa methods by betweenness and PathNet by neighbourhood designs. Further, it could be questioned how well these network measures reflect the real perturbation signals in a pathway.

In the first simulation study we could not identify any PT-based method as outstanding neither for any parameter configuration, nor for the overall accuracy measure. The pathway input for this study was represented by original KEGG pathways, which exhibit considerable gene overlap among each other. When a pathway was called deregulated it received a certain number of genes assigned as affected according to the detection call level. However, a single gene was on average present in 2.4 pathways. This resulted in a number of pathways, which were not considered as deregulated but also contained affected genes. These pathways then showed up as false positives and consequently led to a very low specificity and accuracy of all methods in study 1. However, one could assume that these ‘accidental’ affected genes were allocated randomly in the non-deregulated pathways, whereas in the deregulated pathway they were placed in a topological context. Therefore, it could be expected that the PT-based methods would overcome the problem of these false positives, however, this seems not to be the case.

It was already demonstrated that ranking methods are able to detect modest signals of deregulation better than the ORA approach [20]. We further showed that GS ranking methods were more sensitive than any of the PT-based methods when gene expression changes were weak (*mean* = +/− 1). The CePa GSA method exhibited a clearly different behaviour than the others, which was expected from the single self-contained method included in this work. Prominently different performance of this self-contained method could be further enhance by the fact that CePa computes for each pathway five *p*-values corresponding to different centrality measures and the authors of the method recommend to use the lowest value. Therefore, its overall sensitivity in the first study was higher than all the other competitive methods [12]. On the other hand, its specificity was utterly lost when a lot of pathways were deregulated (study 1, *N* = 70). Similarly, in the diseased benchmark datasets CePa GSA was the best in identifying target pathways but it also identified more than half of all KEGG pathways as significant. Depending on a disease, multiple pathways could be altered. However, it could be questioned whether such a number of pathways is realistic or it reflects lack of specificity of this method.

In the second simulation study with non-overlapping pathways the overall specificity markedly increased, mainly for the PT-based methods. This was also reflected in the best accuracy for these four PT-based methods.

Interestingly, the overall specificity of KS was the lowest among all methods and it was also always decreasing when more genes were affected (see Additional file 4, *DC* = 70 %, *N* = 70, *size* = big). That could stem from its null hypothesis, as it actually tests whether a gene set or its complement is significant [32]. In a setting when it was the complement it resulted in the false positive pathways in our simulations.

Because of the limitations of the simulations, which can never completely capture complex biology, we compared methods also on the real expression data. It is not possible to judge overall accuracy on these 36 benchmark datasets but PathNet and WRS showed reasonable ability to identify the target pathways. This suggests that simple GS approach might be sufficient for detecting enriched pathways.

### Methodological aspects

Several aspects such as pathway data, overlaps of the pathways, and experimental data linked with biological pathways warrant further discussion in the frame of enrichment analysis methodology.

Pathway data stored in the databases are never complete, but regularly curated and updated. To process a new database version into a topological pathway input takes more time and effort than to create the gene sets. Therefore, the PT-based methods are less flexible than GS methods within the respect of pathway data. Further, the true topology of a pathway is context-dependent and differs in between organisms, cell types and tissues [13].

The problem of pathway overlaps has already been pointed out by several authors, including the authors of CePa and PathNet [11, 24] and others [28, 33]. The PathNet method claims to account for the overlap problem by constructing the *pooled pathway.* From the results of study 1 the PT-based approach does not appear to address this problem. However, this might be caused by a limited simulation design. There were further suggestions to overcome the problem of pathway overlaps by testing the subtracted gene sets or the intersects of gene sets [33] or by down-weighting overlapping genes in the analysis [28]. However, these approaches were applied only within the GS methods.

Another issue within the frame of enrichment analyses stems from the mapping of gene expression levels on the pathways, which consist of membrane receptors, intracellular proteins, small molecules, transcription factors and target genes. As biological pathways represent such complex multi-layer models it requires considerable effort to simplify these models into suitable inputs for enrichment algorithms. During this simplification proteins are often replaced by genes. This leads to a discrepancy between experimental data (gene expression profiles) and the levels of the pathways with which they are associated. Therefore, interpretability of results obtained within such an approach is limited, even if the gene expression data does approximate the proteome to a certain extent. Furthermore, if a pathway gene set, used as prior knowledge for the enrichment analysis, does not properly reflect the experimental data, we cannot expect that adding topological information into such a gene set would increase the method’s performance.

A straightforward solution to address this discrepancy would be to test enrichment of signalling pathways in protein abundance data as they reflect the protein character of the pathways much more closely. Another solution might be to move from testing enrichment of pathways to testing sets of target genes of transcription factors, as already proposed by Naeem et al. [34]. However, this requires databases, which collect and curate this type of knowledge. The Broad Institute offers within its Molecular Signatures Database [35] several interesting gene set collections. For instance, the transcription factor targets (C3 TFT) is a collection of sets of genes that share a transcription factor binding site. The chemical and genetic perturbations (C2 CGP) collection represents expression signatures of perturbation experiments. Within the analysis of gene expression data, testing these sets might be more appropriate than testing pathways. Therefore, a gene set does not necessarily have to represent only a pathway. Furthermore, it is easier to handle a gene set in comparison to the topological representation of a pathway. Within the PT-based methods it should also be considered that the pathway topology might be biased or incomplete. Moreover, a pathway in a database is usually given by a certain condition or state, while the experimental data examined in the enrichment analysis comes from biological systems under different states, conditions or disease type. However, visualization of analysed pathways as networks can ease the biological interpretations and reveal further findings of interesting genes and interactions.

Usually, new PT-based methods are evaluated on a small number of real datasets and not in all cases their performance is compared to any other PT-based method [8–11, 24]. We offered one of the first comparative study on PT-based and GS methods, however more comprehensive studies and further benchmark data are needed to systematically evaluate these methods.

## Conclusions

Enrichment analysis is a useful and popular approach to ease interpretation of high throughput data. We comparatively investigated 3 gene set (GS) and 4 pathway topology-based (PT-based) methods in extensive simulation studies and on real benchmark data.

Our results are consistent with some already published findings: As expected, the self-contained method behaved differently than the competitive ones. The ranking methods were more sensitive than over-representation analysis methods when expression changes were moderate. Further, we demonstrated that on original KEGG pathways none of the PT-based methods was clearly outperforming the GS approach, neither in simulations nor in benchmark testing. This changed in the simulation study with non-overlapping pathways, where the PT-based approach exceeded simple GS tests. This suggests that further conceptual work has to be done to deal with the problem of pathway overlaps.

From the gene set methods Wilcoxon rank sum test performed comparably as topological methods in most settings, and it is much easier to use and re-implement with different input data than PT-based approaches.

Including topology to test for enrichment of a pathway is an interesting approach. It introduces additional information into the analysis and allows attractive visualizations of the pathway graphs. However, it needs to be further explored whether the additional biological insights justify the increased complexity of the PT-based enrichment analyses.

## Availability of supporting data

The data sets supporting the results of this article are included within the article and its additional files.

## Additional files

Additional file 1:
**Covariance matrix.** (A) Close positive definite approximation of the covariance matrix for simulation study 1 with original overlapping pathways. (B) Zoom-in of original covariance matrix depicting pathway overlaps: correlation of the genes within one pathway set to 0.8; 0.05 otherwise. (PDF 83 kb) Additional file 2:
**Detailed description of variable parameters.** (DOC 14 kb) Additional file 3:
**KEGG pathway size distribution.** Parsed KEGG databases consisted of 116 pathways, with the smallest pathway of 5 genes and the biggest of 380 genes. Red lines depict 25 % and 75 % quantiles which served as ranges for pathway size parameter. (PDF 30 kb) Additional file 4:
**Specificity in the simulation studies.** Specificity scores of 7 methods under 17 parameter configurations in the simulation study 1 with original pathways with overlapping genes and in the simulation study 2 with non-overlapping pathways with unique gene IDs. Each cell summarizes a median value of 1000 runs. The same colour code key implies for all simulation types. (PDF 135 kb)

## Abbreviations

DEGs: Differentially expressed genes
GS: Gene set
PT-based: Pathway topology based
ORA: Over-representation analysis
FCS: Functional class scoring
KEGG: Kyoto encyclopedia of genes and genomes
WRS: Wilcoxon rank sum test
KS: Kolmogorov-Smirnov test
FE: Fisher’s exact test
FDR: False discovery rate
SPIA: Signaling pathway impact analysis
logFC: Logarithm of fold-change
DC: Detection call
