# Supplementary figures and images for: Comparative study on gene set and pathway topology-based enrichment methods
Source: BMC Bioinformatics. 2015 Oct 22;16:334. doi: 10.1186/s12859-015-0751-5 (PMC4618947; doi:10.1186/s12859-015-0751-5)

**A**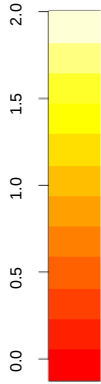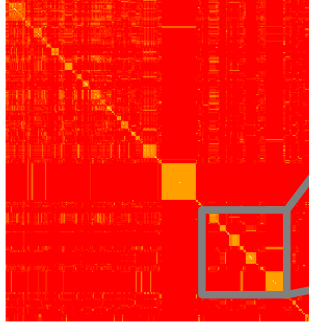**B**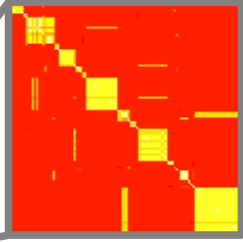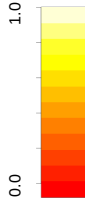

Supplement: Additional file 1: — Covariance matrix. (A) Close positive definite approximation of the covariance matrix for simulation study 1 with original overlapping pathways. (B) Zoom-in of original covariance matrix depicting pathway overlaps: correlation of the genes within one pathway set to 0.8; 0.05 otherwise. (PDF 83 kb) [file 12859_2015_751_MOESM1_ESM.pdf]

# Histogram of size of 116 KEGG pathways

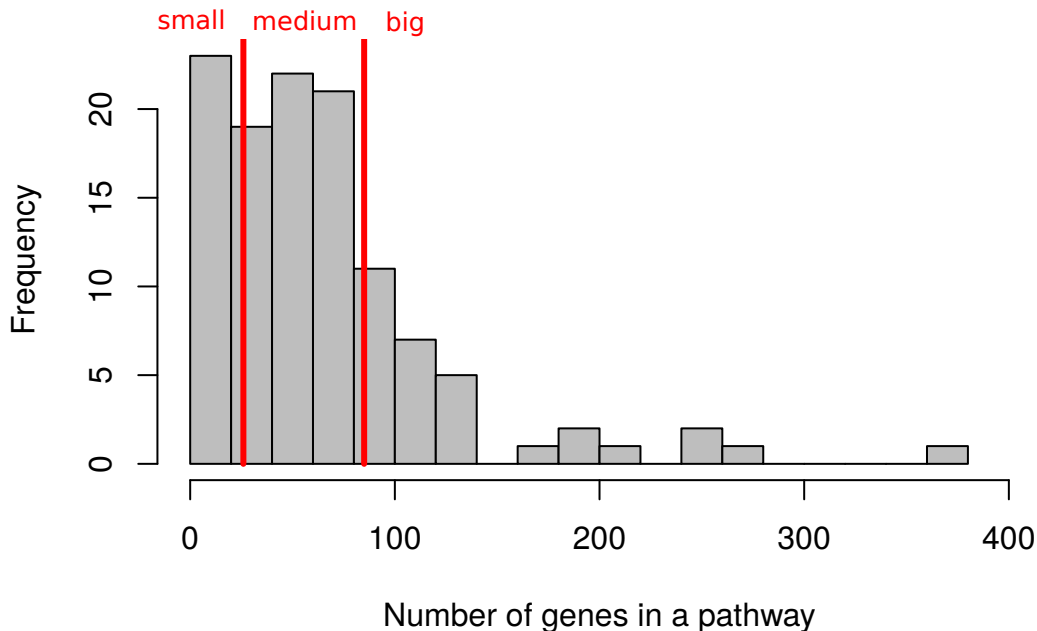

Supplement: Additional file 3: — KEGG pathway size distribution. Parsed KEGG databases consisted of 116 pathways, with the smallest pathway of 5 genes and the biggest of 380 genes. Red lines depict 25 % and 75 % quantiles which served as ranges for pathway size parameter. (PDF 30 kb) [file 12859_2015_751_MOESM3_ESM.pdf]
