# Supplementary material for: Comparative study on gene set and pathway topology-based enrichment methods
Source: BMC Bioinformatics. 2015 Oct 22;16:334. doi: 10.1186/s12859-015-0751-5 (PMC4618947; doi:10.1186/s12859-015-0751-5)
Supplement: Additional file 2: — Detailed description of variable parameters. (DOC 14 kb) [file 12859_2015_751_MOESM2_ESM.doc]

**Detailed description of variable parameters.**

Mean vector: *mean*

To investigate the impact of mean vector changes we choose three levels *mean* = {+/-1, +/-2, +/-6}. These expression changes were introduced for affected genes into the mean vector of the treatment group, while the mean vector of the control group remained always 0. The different magnitude of *mean* change is later reflected in logFC of a gene in between two conditions. The direction of expression change in the *mean* was positive (+) for one half and negative (-) for the second half of the affected genes. When ranges of other parameters were investigated the expression change was set to *mean* = +/-2.

Pathway size: *size*

The second parameter was size of the deregulated pathways *size* = {small, medium, big}. All pathways in our KEGG pathway data input were stratified according to their number of genes into 3 size groups. Small pathways contain from 5 to 26 genes (minimum to 25% quantile), medium pathways have from 27 to 85 genes and big pathways consist of 86 up to 380 genes (75% quantile to maximum) (see Additional file 3). When this parameter was set as fixed, pathways of all sizes were taken into account.

Number of pathways: *N*

Further, we were interested in studying the effect of different proportions of the whole pathway database being deregulated. The number of deregulated pathways *N* = {12, 23, 70} represent approximately 10%, 20% and 60% of all pathways in the KEGG database. Ten percent of all pathways (*N* = 12) were assigned as deregulated when another parameter was variable.

Detection call: *DC*

To explore how many genes in a pathway have to be assigned as affected in order to detect this pathway as significant we examined the percentage of affected genes in a deregulated pathway – the so-called detection call (DC) [31]. Four levels of detection call were investigated: *DC* = {10%, 30%, 50%, 70%} and half of the genes in a deregulated pathway were affected **(***DC* = 50%) when this parameter was not variable in a given simulation type.
