# Supplementary material for: Comparative study on gene set and pathway topology-based enrichment methods
Source: BMC Bioinformatics. 2015 Oct 22;16:334. doi: 10.1186/s12859-015-0751-5 (PMC4618947; doi:10.1186/s12859-015-0751-5)
Supplement: Additional file 4: — Specificity in the simulation studies. Specificity scores of 7 methods under 17 parameter configurations in the simulation study 1 with original pathways with overlapping genes and in the simulation study 2 with non-overlapping pathways with unique gene IDs. Each cell summarizes a median value of 1000 runs. The same colour code key implies for all simulation types. (PDF 135 kb) [file 12859_2015_751_MOESM4_ESM.pdf]

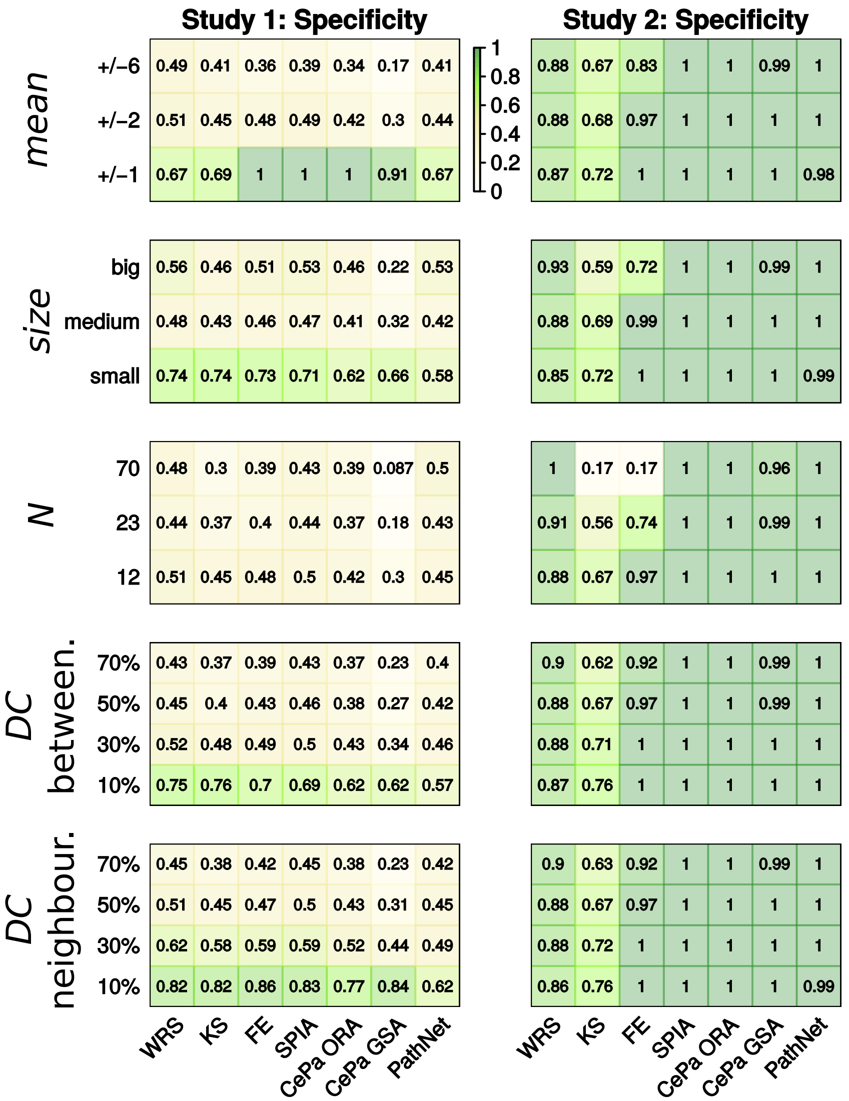
Study 1: Specificity
Study 2: Specificity
mean
+/−6 0.49 0.41 0.36 0.39 0.34 0.17 0.41
+/−2 0.51 0.45 0.48 0.49 0.42 0.3 0.44
+/−1 0.67 0.69 1 1 1 0.91 0.67
+/−6 0.88 0.67 0.83 1 1 0.99 1
+/−2 0.88 0.68 0.97 1 1 1 1
+/−1 0.87 0.72 1 1 1 1 0.98
1
0.8
0.6
0.4
0.2
0
size
big 0.56 0.46 0.51 0.53 0.46 0.22 0.53
medium 0.48 0.43 0.46 0.47 0.41 0.32 0.42
small 0.74 0.74 0.73 0.71 0.62 0.66 0.58
big 0.93 0.59 0.72 1 1 0.99 1
medium 0.88 0.69 0.99 1 1 1 1
small 0.85 0.72 1 1 1 1 0.99
N
70 0.48 0.3 0.39 0.43 0.39 0.087 0.5
23 0.44 0.37 0.4 0.44 0.37 0.18 0.43
12 0.51 0.45 0.48 0.5 0.42 0.3 0.45
70 1 0.17 0.17 1 1 0.96 1
23 0.91 0.56 0.74 1 1 0.99 1
12 0.88 0.67 0.97 1 1 1 1
DC between.
70% 0.43 0.37 0.39 0.43 0.37 0.23 0.4
50% 0.45 0.4 0.43 0.46 0.38 0.27 0.42
30% 0.52 0.48 0.49 0.5 0.43 0.34 0.46
10% 0.75 0.76 0.7 0.69 0.62 0.62 0.57
70% 0.9 0.62 0.92 1 1 0.99 1
50% 0.88 0.67 0.97 1 1 0.99 1
30% 0.88 0.71 1 1 1 1 1
10% 0.87 0.76 1 1 1 1 1
DC neighbour.
70% 0.45 0.38 0.42 0.45 0.38 0.23 0.42
50% 0.51 0.45 0.47 0.5 0.43 0.31 0.45
30% 0.62 0.58 0.59 0.59 0.52 0.44 0.49
10% 0.82 0.82 0.86 0.83 0.77 0.84 0.62
70% 0.9 0.63 0.92 1 1 0.99 1
50% 0.88 0.67 0.97 1 1 1 1
30% 0.88 0.72 1 1 1 1 1
10% 0.86 0.76 1 1 1 1 0.99
WRS
KS
FE
SPIA
CePa ORA
CePa GSA
PathNet
WRS
KS
FE
SPIA
CePa ORA
CePa GSA
PathNet
